# Optimization of Bond Strength Between Heat-Polymerized PMMA and Contemporary CAD/CAM Framework Materials: A Comparative In Vitro Study

**DOI:** 10.3390/polym17111488

**Published:** 2025-05-27

**Authors:** Başak Topdağı

**Affiliations:** Department of Prosthodontics, Faculty of Dentistry, Hamidiye Campus, University of Health Sciences, Istanbul 34668, Türkiye; basak.topdagi@sbu.edu.tr

**Keywords:** polymer composites, CAD/CAM frameworks, surface properties

## Abstract

This study aimed to comparatively evaluate the effects of various surface treatment protocols on the shear bond strength (SBS) between heat-polymerized polymethyl methacrylate (PMMA) and different CAD/CAM framework materials, including cobalt–chromium (Co–Cr) alloys, ceramic particle-reinforced polyetheretherketone (PEEK), and glass fiber-reinforced composite resin (FRC). A total of 135 disc-shaped specimens were prepared from Co–Cr, PEEK, and FRC materials. Surface treatments specific to each material, including airborne-particle abrasion, sulfuric acid etching, laser irradiation, plasma activation, and primer application, were applied. PMMA cylinders were polymerized onto the treated surfaces, and all specimens were subjected to 30,000 thermal cycles. SBS values were measured using a universal testing machine, and the failure modes were classified. The normality of data distribution was assessed using the Kolmogorov–Smirnov test, and the homogeneity of variances was evaluated using Levene’s test. Group comparisons were performed using the Kruskal–Wallis test, and Dunn’s post hoc test with Bonferroni correction was applied in cases where significant differences were detected (α = 0.05). The highest SBS values (~27–28 MPa) were obtained in the Co–Cr group and in the PEEK groups treated with sulfuric acid and primer. In contrast, the PEEK group with additional laser treatment exhibited a lower SBS value. The untreated PEEK group showed significantly lower SBS (~3.9 MPa) compared to all other groups. The Trinia groups demonstrated intermediate SBS values (16.5–17.4 MPa), which exceeded the clinically acceptable threshold of 10 MPa. SEM observations revealed material- and protocol-specific surface responses; plasma-treated specimens maintained topographic integrity, whereas laser-induced surfaces showed localized degradation, particularly following dual-step protocols. Fracture mode analysis indicated that higher SBS values were associated with cohesive or mixed failures. SEM observations suggested that plasma treatment preserved surface morphology more effectively than laser treatment. This study highlights the importance of selecting material-specific surface treatments to optimize bonding between CAD/CAM frameworks and PMMA. Sulfuric acid and primer provided strong adhesion for PEEK, while the addition of laser or plasma offered no further benefit, making such steps potentially unnecessary. Trinia frameworks also showed acceptable performance with conventional treatments. These findings reinforce that simplified conditioning protocols may be clinically sufficient, and indicate that FRC materials like Trinia should be more fully considered for their broader clinical potential in modern CAD/CAM-based prosthetic planning.

## 1. Introduction

With the development of digital technologies in dental practice, possibilities regarding materials used in dental prosthesis fabrication have expanded, and traditional materials and manufacturing methods have been replaced by computer-aided design and computer-aided manufacturing (CAD/CAM) systems [1]. These systems enable the standardized, rapid, and precise fabrication of various framework materials [2]. The biocompatibility, mechanical properties, aesthetics, and bondability of framework materials to veneering materials play a significant role in clinical success [3].

Cobalt–chromium (Co-Cr) alloys, widely used for many years, remain preferred framework materials due to their high modulus of elasticity, long-term durability, and low cost. Despite unresolved disadvantages such as aesthetic limitations and susceptibility to cohesion failures, their compatibility with digital manufacturing techniques, particularly Selective Laser Sintering (SLS), has facilitated their integration into modern applications [4]. Co-Cr materials were included in the study design because they remain popular due to their contemporary production using modern technologies and exhibit strong bonding compatibility with acrylic hybrid prostheses.

As a result of evolving aesthetic perceptions, interest in metal-free alternatives with superior aesthetics is steadily increasing. A high-performance polymer based on polyetheretherketone (PEEK) and reinforced with ceramic particles is used in hybrid prosthesis applications due to its biocompatibility and favorable elasticity [5]. A CAD/CAM composite consisting of a resin matrix reinforced with glass fibers (FRC) stands out with its high fracture resistance, ease of milling, and an elastic modulus similar to that of natural teeth. It is increasingly preferred as an alternative to traditional metals in prosthetic frameworks [6].

The clinical success of prosthetic framework materials depends not only on their mechanical and physical properties but also on the quality of their bonding with the veneering material [7]. Although heat-polymerized PMMA, commonly used in hybrid prostheses, offers aesthetic and biomechanical advantages, achieving strong direct bonding to inert surfaced framework materials remains challenging [8]. Therefore, enhancing the bonding between PMMA and inert framework materials through surface roughening techniques (such as sandblasting or acid etching) and the application of various chemical agents (including primers and bonding agents) can significantly improve the performance of the resulting hybrid structure.

Previous studies have shown that, various surface modification techniques can effectively enhance the bond strength of PEEK and FRC framework materials [9]. Although surface treatments such as airborne-particle abrasion with aluminum oxide (Al_2_O_3_) particles or the sole application of methacrylate-based primers can provide some degree of bonding on PEEK surfaces, these methods offer limited surface activation and generally result in lower bond strength. In contrast, surface treatment with 98% sulfuric acid for 60 s followed by the application of a methacrylate-based primer, or tribochemical silica coating using 110 µm silica coated Al_2_O_3_ particles (2.5 bar pressure, 10 mm distance, 15 s) followed by silanization, have been reported in the literature as the most effective protocols for achieving high bond strength [10,11].

In the literature, various surface treatment protocols have been developed to enhance the bond strength of glass fiber-reinforced composite resins (FRC). These treatments are generally classified into mechanical (physical) roughening methods and chemical surface modifications. Among the most commonly applied surface treatments are airborne-particle abrasion (sandblasting), silane application, plasma surface treatment, and chemical etching techniques such as hydrofluoric acid [12]. Airborne-particle abrasion partially removes the hardened resin matrix layer on the composite surface, thereby exposing the underlying glass fibers. This process increases surface roughness and the available bonding area [13]. Silane application can improve bonding by chemically modifying glass particles; however, it is generally insufficient alone. Laser and plasma treatments enhance surface energy and wettability by introducing oxygen-containing functional groups [14,15]. Numerous studies have shown that combining mechanical roughening with chemical conditioning yields the highest bond strength, as mechanical treatments create micromechanical retention while chemical agents enhance surface chemistry to improve adhesion [16].

This study aims to comparatively evaluate the effects of different surface treatment protocols on the bond strength between heat-polymerized PMMA and contemporary framework materials, and the associated fracture types. Additionally, the morphological effects of these surface treatments will be analyzed using scanning electron microscopy (SEM) for surface characterization.

To the best of our knowledge, no previous studies have simultaneously evaluated and directly compared the bond strength between heat-polymerized PMMA and three contemporary CAD/CAM framework materials—Co-Cr alloys, ceramic-reinforced PEEK, and fiber-reinforced composite (Trinia)—using standardized surface treatment protocols. Although standalone laser or plasma treatments alone are insufficient compared to chemical surface treatments such as acid etching or priming on PEEK surfaces, the potential synergistic effect of combining these physical methods (laser/plasma) with chemical pretreatments remains unclear [17,18]. Additionally, the literature regarding surface conditioning protocols for Trinia (FRC) is notably limited, underscoring a significant gap in knowledge [12]. This study provides a comprehensive and comparative evaluation of a material that, despite its increasing clinical relevance, remains significantly underexplored in the literature. Beyond simply repeating conventional protocols, we systematically introduced novel surface roughening strategies—namely the combination of plasma and laser with chemical conditioning—to explore their potential synergistic effects. In doing so, the study not only addresses a long-standing gap in the in vitro validation of fiber-reinforced CAD/CAM composites like Trinia, but also proposes a surface optimization approach not previously reported. Ironically, while CAD/CAM-based workflows are increasingly celebrated as the future of prosthodontics, the foundational materials enabling these systems—such as Trinia—have not received the systematic investigation they deserve. Without robust, evidence-based data on the bonding performance of such frameworks, the promise of CAD/CAM remains conceptually impressive but clinically incomplete.

In the present study, two hypotheses are proposed. The first hypothesis suggests that, for high-performance polymer-based framework materials, incorporating plasma and laser applications into surface treatment protocols recognized in the literature as the most effective for maximizing shear bond strength (SBS) will result in a significant enhancement of bond strength. The second hypothesis posits that the shear bond strength between glass fiber-reinforced composite (FRC) and polymethyl methacrylate (PMMA) can reach a clinically acceptable level when appropriate surface treatment methods are employed.

## 2. Material and Methods

This study was conducted exclusively on industrially manufactured framework materials (Co-Cr, PEEK, and glass fiber-reinforced composite resin) and did not involve any human or animal specimens. Therefore, ethical approval was not required. A priori power analysis was performed using G*Power (v3.1.9.7) for a one-way ANOVA with 9 groups (α = 0.05, power = 0.80, effect size f = 0.40). The analysis indicated that a minimum of 12 samples per group was required. To account for potential data loss, 15 samples per group were included, yielding a total sample size of 135. Three different framework materials were evaluated in this study: cobalt–chromium (Co–Cr) metal alloy (Wirobond^®^ C+, BEGO Medical GmbH, Bremen, Germany), polyetheretherketone (BioHPP^®^, Bredent GmbH, Senden, Germany), and glass fiber-reinforced CAD/CAM composite (Trinia^®^, Bicon LLC, Boston, MA, USA).

### 2.1. Specimen Preparation

A total of 135 disc-shaped specimens, each with a diameter of 10 mm and a thickness of 2 mm, were prepared in accordance with ISO 4049 [19] framework and the recent literature to ensure dimensional consistency and comparability in polymer-based bond strength testing. Three different framework materials were evaluated in this study: cobalt–chromium (Co–Cr) metal alloy (Wirobond^®^ C+, BEGO Medical GmbH, Bremen, Germany), polyetheretherketone (BioHPP^®^, Bredent GmbH, Senden, Germany), and glass fiber-reinforced CAD/CAM composite (Trinia^®^, Bicon LLC, Boston, MA, USA). A total of 135 disc-shaped specimens, each with a diameter of 10 mm and a thickness of 2 mm, were prepared. The metal specimens were fabricated using direct metal laser sintering (DMLS) with BEGO Mediloy^®^ S-Co alloy powder in an EOS M100 system (EOS GmbH, Krailling, Germany), employing an additive manufacturing approach with a layer thickness of 20 µm [20]. A layer thickness of 20 µm was employed in accordance with the standard parameter sets recommended by the manufacturer for the EOS M100 system when processing Mediloy^®^ S-Co alloy powder, ensuring optimal surface quality and mechanical properties. The BioHPP and Trinia specimens were milled from prefabricated CAD/CAM blocks using a computer numerical control (CNC) unit (Ceramill Motion 2, Amann Girrbach AG, Koblach, Austria) in accordance with the manufacturers’ guidelines [21].

After fabrication, specimen surfaces were cleaned in an ultrasonic bath containing 96% ethanol for 10 min to remove surface shavings and manufacturing residues in accordance with ISO 10993-12 [22] guidelines for sample preparation. The cleaned specimens were stored in sterile containers and protected from light until random allocation to surface treatment groups. Each specimen was individually coded before the procedure. All procedures were performed by the same operator under constant ambient temperature and humidity to ensure methodological consistency.

### 2.2. Application of Surface Treatments

The specimens were randomly allocated into predefined groups within each material type, based on surface treatment protocols. Surface treatments were applied using literature-supported protocols selected according to the physical and chemical properties of each material. The commercial names, manufacturers, and product codes of all materials and surface treatment agents are presented in Table 1. A summary of the mechanical and surface properties of the tested framework and veneering materials is provided in Table 2.

The classification and corresponding codes of all experimental groups, based on the applied surface treatment protocols for each material, are detailed in Table 2. A total of 9 experimental groups were established, each consisting of 15 specimens (*n* = 15), resulting in 135 specimens overall (Table 3).

The surfaces of the Co–Cr specimens (Group MG) were airborne-particle abraded using 110 µm aluminum oxide (Al_2_O_3_) particles at a pressure of 2 bar. Abrasion was performed perpendicularly from a 10 mm distance for 10 s. Following abrasion, the surfaces were cleaned with oil-free compressed air. A metal primer (Scotchbond Universal; 3 M ESPE, St. Paul, MN, USA) was then applied according to the manufacturer’s instructions and allowed to react for 180 s.

In the PEP group, the surfaces of the BioHPP specimens were treated with 98% sulfuric acid (Merck; Sigma-Aldrich, Saint Louis, MO, USA) for 60 s to promote chemical activation. The specimens were then thoroughly rinsed with distilled water (Aqua Dist; Arzerum Kimya, Istanbul, Türkiye) and air-dried prior to bonding. In the PEP-L group, a Ytterbium-doped fiber laser (1064 nm; YLP Series, IPG Photonics Corporation, Oxford, MA, USA) was applied for 30 s prior to acid application in order to induce micromechanical surface modification. Following laser treatment, 98% sulfuric acid was applied for 60 s. The specimens were subsequently rinsed and dried as described for the PEP group. In the PEP-PL group, surface treatment consisted of 98% sulfuric acid etching for 60 s, followed by plasma activation to enhance surface energy. Plasma treatment was performed for 60 s under atmospheric pressure using the PlasmaPrep III system (IPG Photonics Corporation, Oxford, MA, USA). After each procedure, specimens were rinsed with distilled water and air-dried.

In the TCP group, a tribochemical surface treatment was performed using 30 µm silica-coated aluminum oxide particles (CoJet™ Sand; 3M ESPE, Bayern, Germany) at 2 bar pressure, applied perpendicularly from a 10 mm distance for 10 s. The specimens were then cleaned with oil-free compressed air. In the TAP group, surface roughening was achieved by sandblasting with 50 µm aluminum oxide particles (Cobra; Renfert GmbH, Hilzingen, Germany) under 2 bar pressure, applied perpendicularly from a distance of 10 mm for 10 s. After sandblasting, the specimens were cleaned using oil-free compressed air. In the TAP-L group, the specimens were first irradiated with a Ytterbium-doped fiber laser (1064 nm; YLP Series, IPG Photonics Corporation, Oxford, MA, USA) for 30 s to induce micromechanical surface modification. This was followed by sandblasting as described for the TAP group. In the TAP-PL group, sandblasting was performed as in the TAP group, followed by plasma activation for 60 s under atmospheric pressure using the PlasmaPrep III system (IPG Photonics Corporation, Oxford, MA, USA) to enhance surface energy

All procedures were performed by a single operator under controlled ambient temperature (23 ± 1 °C). The surfaces of the specimens were inspected before and after each procedure according to standardized protocols. The prepared specimens were stored in a dust-free and light-protected environment until the bonding stage.

### 2.3. SEM Analysis

To evaluate the morphological alterations induced by surface treatments on the framework materials, one representative specimen was randomly selected from each laser- and plasma-treated group for examination under a scanning electron microscope (FE-SEM, Gemini 500, Carl Zeiss, Oberkochen, Germany). Imaging was performed using an acceleration voltage consistent with the manufacturer’s guidelines, and the specimens were sputter-coated with gold to ensure conductivity. SEM images were acquired at 10,000× magnification to document the general surface topography. Additionally, for groups subjected to combined or high-energy surface treatments, SEM images were also acquired at 20,000× magnification to evaluate matrix degradation, fissure formation, and microstructural alterations in greater detail. Throughout the imaging process, a standardized protocol was followed by maintaining a constant focal distance and an appropriate working distance.

### 2.4. Preparation of Heat-Polymerized PMMA Specimens

To fabricate the veneering specimens, heat-polymerized polymethyl methacrylate (PMMA) material (Trevalon; Dentsply Sirona, Gurgaon, India) was used. Disc-shaped metal molds with a diameter of 10 mm and a thickness of 2 mm were employed to ensure dimensional standardization and compatibility with the underlying specimens, in accordance with ISO 4049 [19] guidelines for polymer-based dental materials. The PMMA material was manually mixed according to the manufacturer’s instructions and packed into the molds. Polymerization was carried out in a pressurized polymerization unit (Ivomat; Ivoclar Vivadent AG, Schaan, Liechtenstein) at 100 °C and 6 bar pressure for 20 min. Following polymerization, the molds were carefully removed, and each specimen was examined using a digital caliper to verify dimensional accuracy. The fabricated PMMA discs were stored at room temperature in closed containers until surface treatment and bonding procedures were performed.

### 2.5. PMMA Surface Treatment and Bonding Protocol

The fabricated heat-polymerized PMMA discs were visually inspected, and 135 specimens with intact, defect-free surfaces were selected. The bonding surfaces of each disc were air-abraded using 50 µm aluminum oxide particles (Cobra; Renfert GmbH, Hilzingen, Germany) under 2 bar pressure, applied perpendicularly from a distance of 10 mm for 10 s. After air abrasion, the specimens were cleaned with oil-free compressed air. To enhance chemical bonding, a thin layer of MMA-based bonding agent (Visio.Link; Bredent GmbH & Co. KG, Senden, Germany) was applied to the bonding surfaces of both the PMMA and framework discs. Polymerization was performed while the discs were positioned in close contact within a standardized index mold (Elite HD+ Putty; Zhermack, Rovigo, Italy), under gentle manual pressure. While held in position, the specimens were light-cured for 90 s using a LED curing unit (Bluephase N; Ivoclar Vivadent AG, Schaan, Liechtenstein). All bonding procedures were conducted in accordance with the surface treatment protocols specific to each material group.

### 2.6. Aging Protocol

All bonded specimens underwent thermocycling to simulate long-term intraoral conditions. A total of 30,000 cycles were applied between 5 °C and 55 °C, with 30 s of immersion at each temperature and a 5 s transfer interval. This protocol was selected to represent the thermal fluctuations occurring over approximately five years of clinical function. Thermocycling was carried out using a programmable device (Thermocycler THE-1100; SD Mechatronik GmbH, Feldkirchen-Westerham, Germany). Specimens were inspected periodically during the process for any macroscopic signs of damage. Upon completion of aging, they were stored in distilled water at 37 °C until testing.

### 2.7. Shear Bond Strength Test

Shear bond strength testing was performed using a universal testing machine (Model 6800; Instron Corp., Norwood, MA, USA). Each bonded specimen consisted of a PMMA disc (10 mm diameter × 2 mm thickness) attached to a framework material. The specimens were placed in a custom-made flat metal fixture to ensure a stable and standardized position during loading. A shear load was applied at a crosshead speed of 1 mm/min, with the direction of force aligned parallel to the bonding interface. Load was applied to the edge of the PMMA disc until debonding occurred. The maximum load (N) was recorded and converted to shear bond strength (MPa) by dividing by the bonded area. All measurements were conducted using the same device and operator under calibrated test conditions. After debonding, failure modes were examined visually and classified as adhesive, cohesive, or mixed.

Following bond strength testing, all specimens were macroscopically evaluated to determine the mode of failure. Fracture surfaces were classified into three main categories—adhesive, cohesive, and mixed—based on definitions commonly cited in the literature. Assessments were performed under fixed-angle lighting, using direct visual inspection and, when necessary, a handheld magnifier (2–3×, AL2492; Microcase, Izmir, Türkiye). All evaluations were conducted by a single blinded examiner to ensure consistency. The identified failure types were recorded and coded for statistical analysis. This assessment provided qualitative insight into bond integrity, offering complementary information regarding the effectiveness of the applied surface treatments.

### 2.8. Statistical Analysis

Statistical analyses were conducted using IBM SPSS Statistics version 26 (IBM Corp., Armonk, NY, USA). The normality of the data distribution was assessed using the Kolmogorov–Smirnov test, and Levene’s test was used to evaluate the homogeneity of variances. As both assumptions were violated (*p* < 0.05), the non-parametric Kruskal–Wallis test was used to compare shear bond strength (SBS) values among the groups. Following a statistically significant overall result, Dunn’s post hoc test with Bonferroni correction was applied for pairwise comparisons. The distribution of failure modes (adhesive, cohesive, mixed) was analyzed using the Pearson Chi-square test. A statistically significant difference was detected among the groups (χ^2^(16) = 52.259, *p* < 0.001), suggesting that the failure mode was affected by the surface treatment protocol. Descriptive crosstabulations were also generated. A *p*-value of less than 0.05 was considered statistically significant throughout the analyses.

## 3. Results

**Figure 1:**
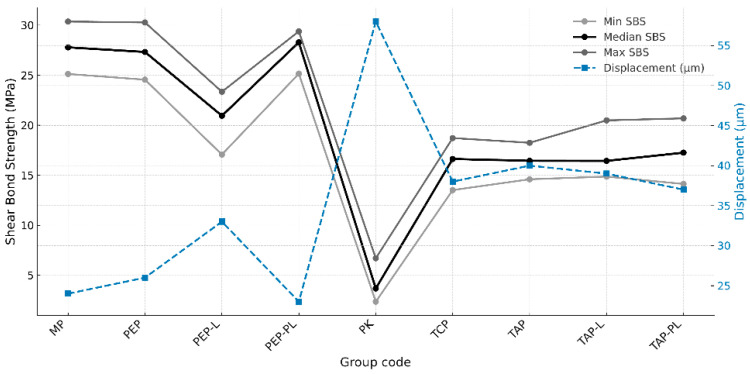
Minimum, median, and maximum shear bond strength (SBS) values (MPa) of all experimental groups following surface treatment and thermal cycling.

The minimum, median, and maximum shear bond strength (SBS) values of the experimental groups are presented in Figure 1. The highest median SBS values were observed in the MP (27.79 MPa), PEP-PL (28.29 MPa), and PEP (27.33 MPa) groups. In contrast, the lowest median value was recorded in the PK group (3.68 MPa). Moderate SBS values were reported in the T-K (16.63 MPa), TAP (16.50 MPa), TAP-L (16.44 MPa), and TAP-PL (17.26 MPa) groups. The PEP-L group, with a lower median value (20.95 MPa), was positioned between the higher and lower SBS level groups (Figure 1).

The Kruskal–Wallis test revealed a statistically significant difference in SBS values among the groups (*p* < 0.05). Post hoc analysis indicated that the PK group exhibited significantly lower bond strength compared to all other groups (*p* < 0.05). The MP and PEP-PL groups showed significantly higher SBS values than the PK, PEP-L, T-K, TAP, TAP-L, and TAP-PL groups (*p* < 0.05). The PEP-L group demonstrated significantly lower SBS values compared to the MP, PEP, and PEP-PL groups (*p* < 0.05). No statistically significant differences were found among the T-K, TAP, TAP-L, and TAP-PL groups (*p* > 0.05). Detailed pairwise comparison results are presented in Table 4.

**Figure 2:**
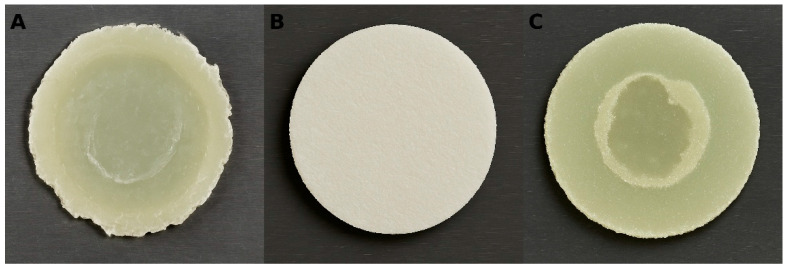
Representative stereomicroscopic images of fracture types observed after shear bond strength testing. (**A**) Adhesive failure at interface between PEEK substrate and polymer. (**B**) Cohesive failure within polymer material. (**C**) Mixed cohesive failure involving both polymer and PEEK substrate. Images were captured under 20× magnification using a stereomicroscope.

Fracture types were evaluated under a stereomicroscope at 20× magnification (Stemi DV4, Zeiss Mikroskopie; Göttingen, Germany) and classified as follows: (1) adhesive failure between the PEEK substrate and the polymer; (2) cohesive failure within the polymer only; (3) cohesive failure within the PEEK substrate only; and (4) cohesive failure involving both the polymer and the PEEK substrate. Representative images of these fracture types are presented in Figure 2.

**Figure 3:**
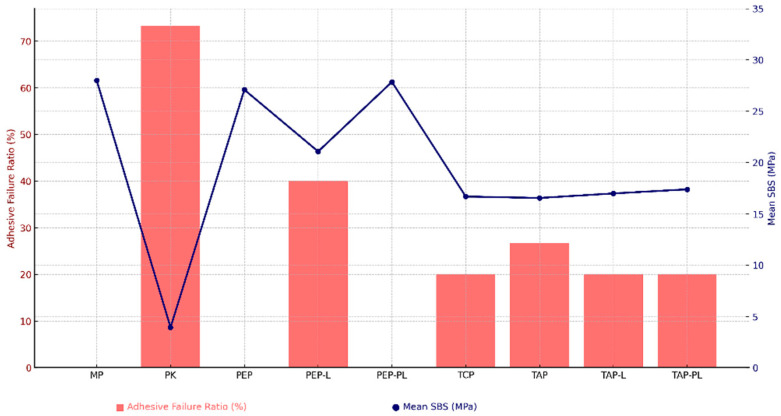
Relationship between adhesive failure ratios and mean shear bond strength (SBS) values for each experimental group.

The distribution of fracture types among the groups is presented in Figure 3. The Pearson Chi-square test revealed a statistically significant difference in fracture mode distribution among the groups (*p* < 0.05). Adhesive failures were predominantly observed in the PK and PEP-L groups, whereas cohesive failures were more frequent in the MP, PEP, and PEP-PL groups. In the T-K, TAP, TAP-L, and TAP-PL groups, the fracture mode distribution was more balanced, with a noticeable proportion of mixed failures. These findings suggest that the surface treatment protocols significantly influenced the integrity and characteristics of the bonding interface. Figure 3 illustrates the relationship between adhesive failure ratios and the corresponding mean SBS values of the experimental groups. Groups with higher SBS values tended to exhibit lower adhesive failure ratios, reinforcing the role of effective surface treatments in promoting cohesive or mixed failures and improving the integrity of the bonding interface.

To assess the morphological impact of the applied surface treatments on polymer-based frameworks, scanning electron microscopy (SEM) analysis was performed on representative specimens from each group. Preliminary evaluations revealed that surface-specific features such as topographic roughness, particle embedding, and fiber exposure became distinguishable at 10,000× magnification. However, in groups subjected to complex multi-step treatments, detailed assessment of matrix degradation, localized melting, and microcrack formation required higher resolution. At 20,000× magnification, these microstructural alterations were more clearly visualized, allowing for a more reliable interpretation of surface integrity.

**Figure 4:**
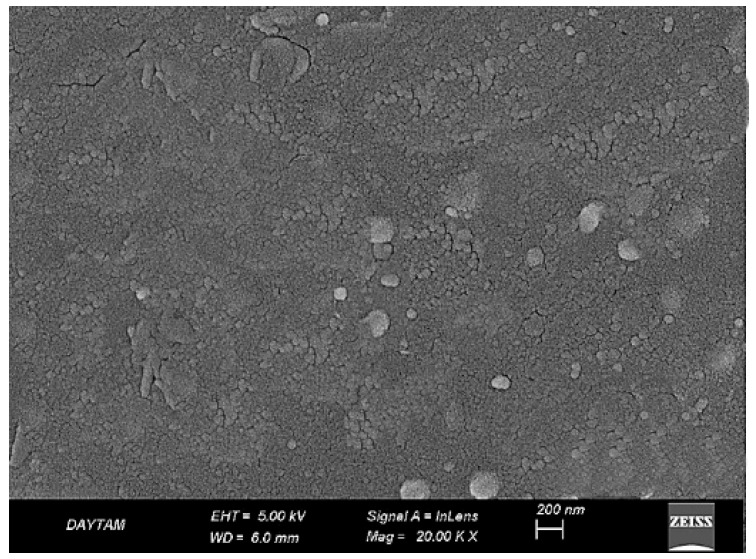
SEM image of untreated BioHPP surface at ×20,000 magnification. A smooth and morphologically intact surface is observed, with no signs of microstructural damage or mechanical roughening.

In the untreated BioHPP specimen (PK20, ×20,000), the surface exhibited an overall intact and continuous morphology with no evidence of structural degradation or matrix disruption. Despite the absence of topographic damage, the surface appeared notably smooth and lacked any significant micromechanical roughness, which may limit adhesive potential in its native state (Figure 4).

**Figure 5:**
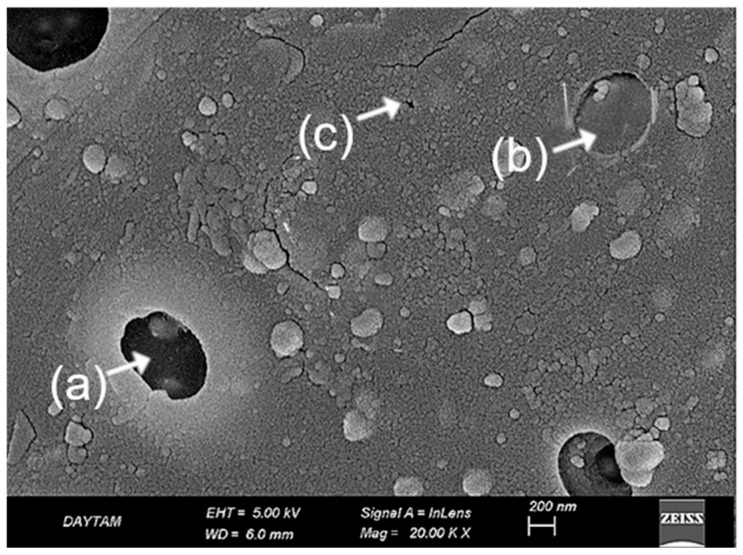
SEM image of sulfuric acid-treated BioHPP surface at ×20,000 magnification. (a) Submicron-scale pits resulting from amorphous layer degradation; (b) acid-induced porosities indicating enhanced surface activation; (c) irregular erosion patterns and matrix softening suggest partial loss of microscale uniformity. White arrows mark key surface features. Scale bar: 1 µm.

SEM analysis of the sulfuric acid-treated BioHPP specimen (Figure 5) revealed pronounced acid-etched porosities and irregular pit structures indicative of extensive surface erosion. These submicron recesses result from the degradation of the amorphous surface layer, increasing the reactive surface area for potential bonding. However, localized softening of the matrix and loss of surface uniformity were also evident, suggesting that the etching protocol may compromise structural homogeneity at the microscale (Figure 5).

**Figure 6:**
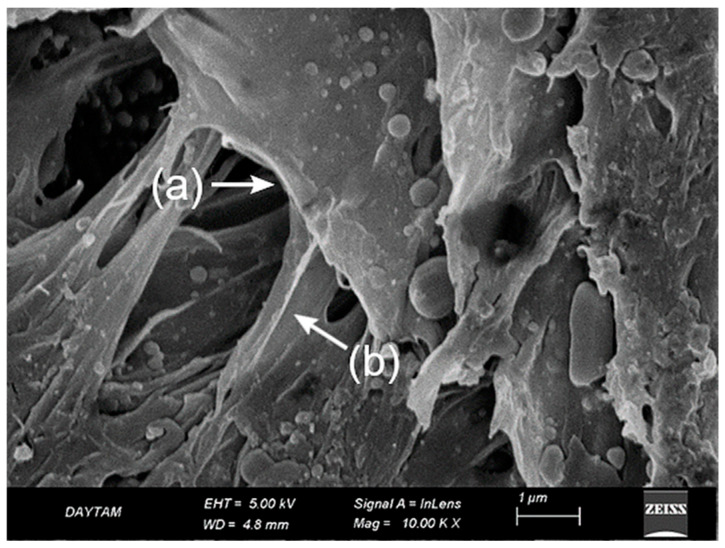
SEM image of BioHPP surface treated with sulfuric acid, primer, and laser at ×10,000 magnification. (a) Local melting of surface due to thermal effects; (b) Flow marks indicating material displacement; partially collapsed pits and surface irregularities are also visible. White arrows mark key features. Scale bar: 1 µm.

In the laser-applied BioHPP specimens, previously acid-etched surface patterns appeared disrupted due to thermal effects. At 10,000× magnification, localized melting, softened pit contours, and flow-like features were observed, suggesting reflow of the polymer matrix. The surface showed inconsistent morphology, with heterogeneous microretentive potential due to irregular deformation zones (Figure 6).

**Figure 7:**
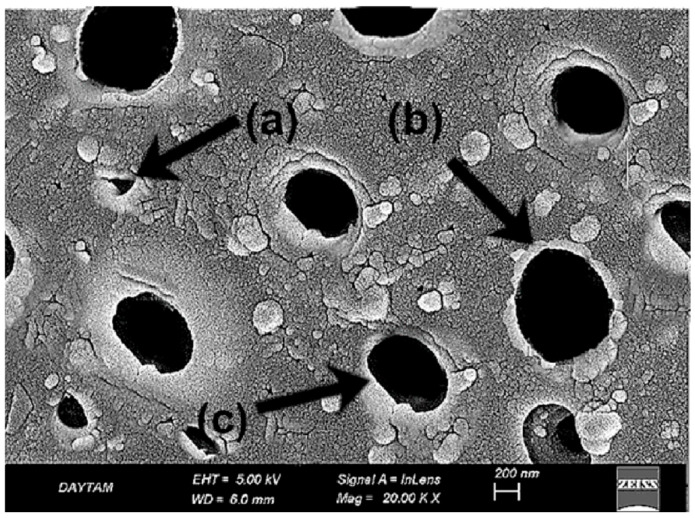
SEM image of same group at ×20,000 magnification. (a) Fused pits with indistinct boundaries; (b) matrix deformation visible as blurred and irregular regions; (c) contraction rings indicating localized shrinkage. Black arrows indicate characteristic morphological features. Scale bar: 1 µm.

At 20,000× magnification these alterations were more clearly delineated. Fused pit borders, amorphous matrix zones, and concentric shrinkage rings indicated further breakdown of microstructural continuity. The addition of laser treatment to an already etched surface led to more pronounced surface erosion and compromised morphological integrity (Figure 7).

**Figure 8:**
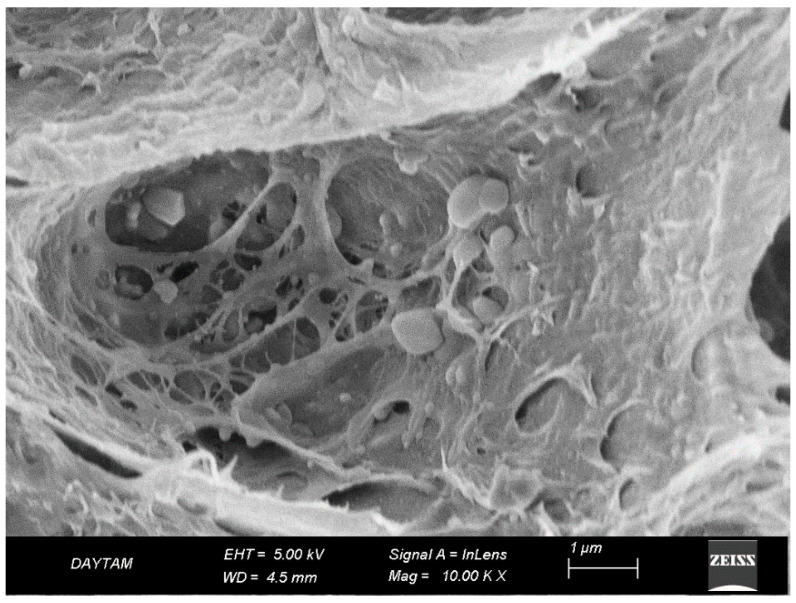
SEM image of BioHPP surface treated with sulfuric acid, primer, and plasma at ×10,000 magnification. A nano-textured surface with uniform shallow dimpled features is observed, without signs of thermal deformation.

The plasma-treated BioHPP surfaces exhibited a uniform and structurally preserved morphology, distinct from the deeper recesses observed in the sulfuric acid group and the thermal degradation noted in the laser group. At 10,000× magnification (Figure 8), nano-scale dimpled and ridged surface patterns were observed, indicating controlled ionic bombardment zones associated with plasma-induced surface activation.

**Figure 9:**
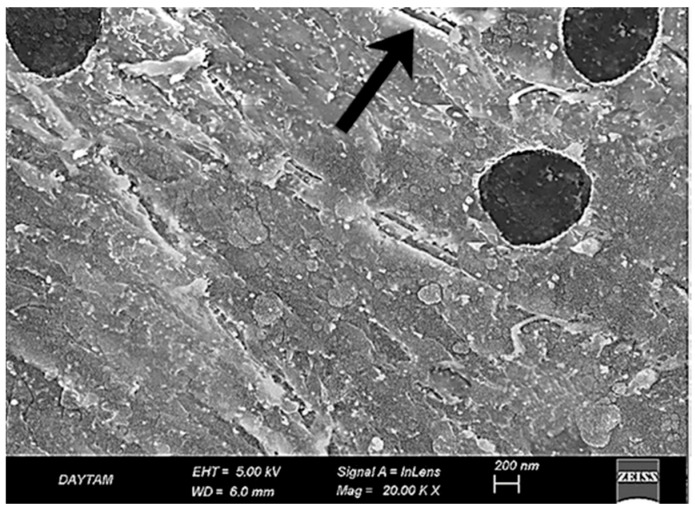
SEM image of same group at ×20,000 magnification. Plasma-induced ravine-like nano-topography is more evident, with preserved matrix continuity and no fiber or resin damage. A representative ravine-like structure is indicated with a black arrow.

At 20,000× magnification (Figure 9), these features became more clearly defined, revealing shallow ravine-like surface irregularities without evidence of matrix melting or fiber deformation. The absence of thermal damage and the preservation of microstructural continuity emphasized the non-invasive nature of plasma modification. Compared to the acid-etched surfaces, plasma treatment produced a more superficial but highly uniform topography, enhancing surface energy without compromising morphological integrity (Figure 9).

**Figure 10:**
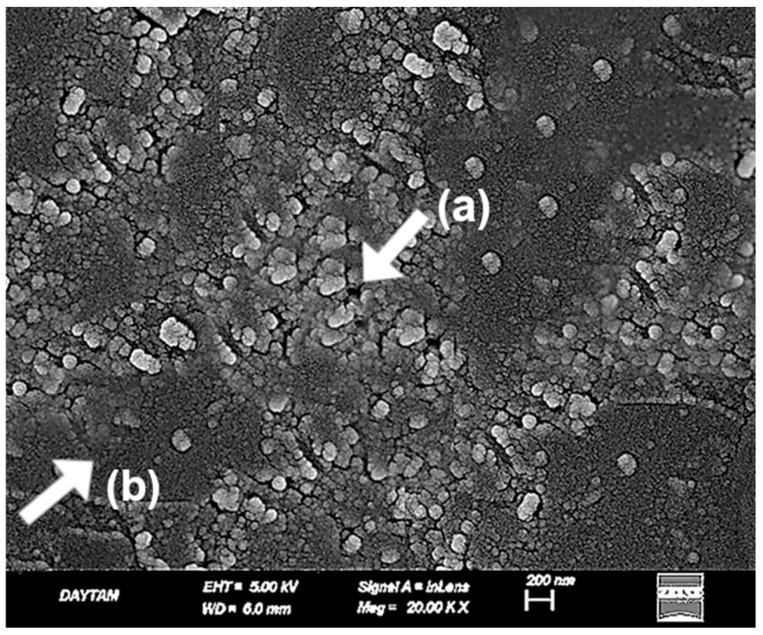
SEM image of Trinia surface after CoJet treatment at ×20,000 magnification. (a) Shallow micro-pits formed by silica particle impact; (b) smoothened matrix regions with no visible fiber exposure or structural damage. White arrows mark the key features. Scale bar: 1 µm.

In the TCP group (Figure 10), CoJet application produced scattered micro-pits and localized surface irregularities due to the impact of silica-coated glass particles. The fiber architecture remained mostly embedded beneath the surface, and the resin matrix showed minimal damage. The resulting surface appeared moderately activated, with limited micromechanical interlocking potential and preserved topographic continuity (Figure 10).

**Figure 11:**
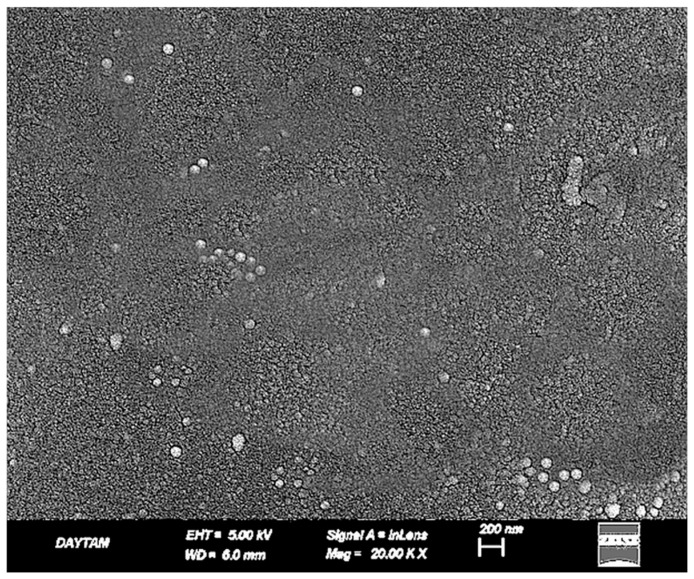
SEM image of Trinia surface after airborne-particle abrasion with aluminum oxide at ×20,000 magnification. Disrupted resin matrix and irregular surface roughness are evident, indicating the mechanical impact of abrasive particles. Scale bar: 200 nm.

In contrast, the TAP group (Figure 11) demonstrated a more aggressive surface profile following airborne-particle abrasion with aluminum oxide. Fiber outlines were clearly exposed, and the resin matrix appeared disrupted and withdrawn in some areas. The generated roughness was deeper and more irregular, offering increased micromechanical retention potential at the expense of microstructural integrity (Figure 11).

**Figure 12:**
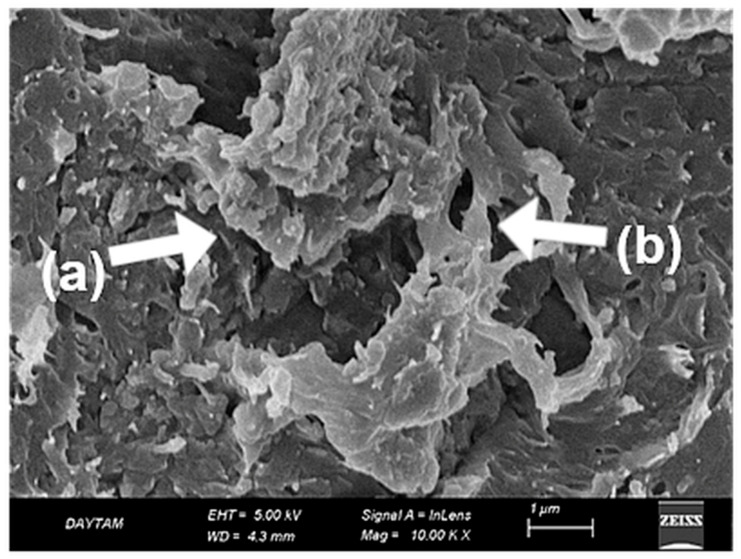
SEM image of Trinia surface after airborne-particle abrasion and laser treatment at ×10,000 magnification. (a) Partial matrix melting with evidence of flow deformation; (b) disrupted fiber–matrix interface with loss of continuity. White arrows indicate representative morphological features. Scale bar: 1 µm.

In the TAP-L group, the laser-modified surfaces exhibited a more complex morphology compared to both TAP and TCP groups. At 10,000× magnification (Figure 12), partial fiber deformation, superficial matrix melting, and loss of topographic continuity were observed. The fiber contours initially exposed by airborne-particle abrasion appeared partially smoothed or distorted due to the thermal effect of the laser, resulting in a non-directional and irregular surface appearance (Figure 12).

**Figure 13:**
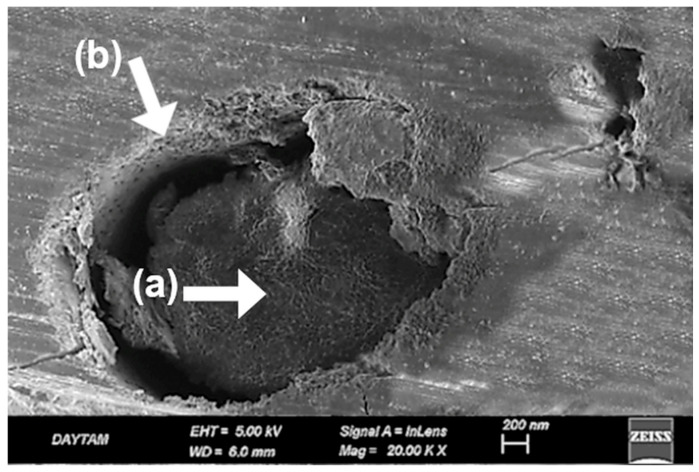
SEM image of same group at ×20,000 magnification. (a) Laser-induced amorphous matrix zones characterized by smooth, glassy surface texture; (b) micro-shrinkage features appearing as localized depressions or contraction rings. White arrows indicate key morphological features. Scale bar: 1 µm.

At 20,000× magnification (Figure 13), these changes were more pronounced. Localized shrinkage zones, amorphous regions within the matrix, and melted fiber edges became evident. The high-energy laser interaction on an already roughened substrate appeared to compromise the previously established micro-retentive architecture. Compared to the TAP group, which displayed a mechanically retentive but structurally stable surface, TAP-L presented a less defined and potentially less reliable bonding interface (Figure 13).

When compared to the TCP group, the TAP-L surface showed deeper roughness but also greater microstructural disruption. These findings suggest that combining multiple surface treatments may result in over-processing, leading to reduced topographic control and compromised surface integrity.

**Figure 14:**
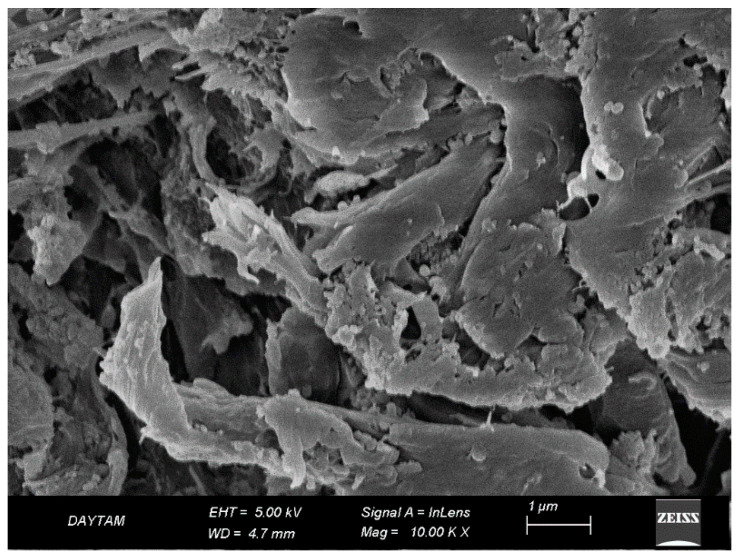
SEM image of Trinia surface after airborne-particle abrasion and plasma treatment at ×10,000 magnification. Surface shows preserved micro-roughness with localized smoothing and refined texture.

**Figure 15:**
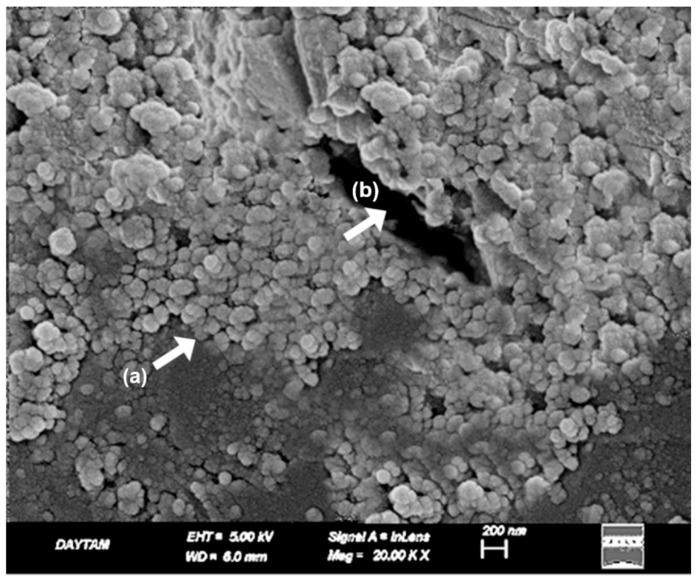
SEM image of same group at ×20,000 magnification. (a) Nano-scale topographic patterns formed by plasma–material interaction; (b) shallow fissure-like features indicating localized degradation. White arrows indicate representative morphological features. Scale bar: 1 µm.

Following plasma treatment, the TAP-PL group retained its overall surface roughness while exhibiting localized smoothing and nano-scale refinement. At both 10,000× (Figure 14) and 20,000× (Figure 15) magnifications, the topography induced by alumina abrasion appeared more homogenized without evident structural degradation. Compared to TAP and TAP-L, the surface showed a more balanced distribution of active regions, while offering deeper and more consistent roughness than the TCP group. At higher magnification, certain regions exhibited fissure-like features that were not visible at lower resolution, suggesting that the second surface treatment (plasma) may have contributed to localized degradation. A similar phenomenon was previously observed in the PEEK-based groups where dual treatments also led to increased surface breakdown (Figure 13 and Figure 15).

## 4. Discussion

This study investigated how different surface treatment protocols affect the shear bond strength (SBS) between high-performance polymer-based frameworks and polymethyl methacrylate (PMMA). The first hypothesis—that incorporating plasma or laser into established treatments would improve SBS—was not supported. In fact, these additions sometimes weakened the bonding interface. The second hypothesis, proposing that certain surface treatments could achieve clinically acceptable SBS levels between glass fiber-reinforced composites (FRC) and PMMA, was confirmed. Overall, the results emphasize the importance of choosing the right treatment combination and sequence to ensure durable bonding in hybrid prosthetic designs.

The shear bond strength (SBS) test was chosen for its reproducibility and its ability to approximate functional loading conditions in the oral cavity, making it a widely accepted method in adhesive dentistry [23].

In hybrid prosthetic applications, the quality of the bond between the framework and veneering materials is essential for biomechanical stability and long-term success. Therefore, in this study, airborne-particle abrasion followed by metal primer application on a cobalt–chromium (Co–Cr) framework—one of the most widely reported and clinically preferred protocols—was selected as the gold standard reference [24]. In previous studies, this protocol has yielded average SBS values between 25 and 30 MPa, especially in pre-aging conditions. Cohesive and mixed failures frequently reported in these studies reflect a strong and durable bond. Although aging may cause a slight reduction, SBS values typically remain well above the clinically acceptable threshold of 10 MPa [25,26]. In the present study, the Co–Cr group treated with airborne-particle abrasion and metal primer (MP) showed an SBS value of 27.8 ± 2.6 MPa, in line with previous findings. Predominantly cohesive failures within the PMMA were also consistent with earlier reports [27,28].

Given PEEK’s low surface energy and chemical inertness, surface treatments are necessary to improve bonding. As in previous studies, a non-treated group was included to serve as a baseline for evaluating the effectiveness of each protocol. In contrast, no untreated control group was included for Trinia, as its unmodified use does not reflect clinical practice. This design choice aimed to generate data that more accurately represent clinical conditions. The most established surface treatment for PEEK involves etching with 98% sulfuric acid followed by the application of an MMA-based primer. In this study, this gold-standard protocol was not replaced, but deliberately challenged by integrating laser and plasma treatments to push its limits. While numerous studies have explored surface conditioning of PEEK, few have sought to refine proven methods with such precision, making the present approach both clinically relevant and scientifically progressive [29]. Subsequent application of a primer containing MMA, PETIA, and dimethacrylate enhances chemical adhesion in addition to the micromechanical interlocking provided by surface roughening [30]. In the present study, additional laser and plasma treatments were applied to further enhance the most established surface conditioning protocol comprising acid etching and primer application. In the PK group, where only the primer was applied without prior acid etching, four out of fifteen specimens exhibited complete interfacial debonding between PEEK and PMMA following mechanical aging. The shear bond strength (SBS) values measured in the remaining specimens were significantly lower than those of all other experimental groups and fell below the clinically acceptable threshold of 10 with a mean value of 3.68 ± 2.1 MPa (*p* < 0.05) [31]. All failures observed in this group were of the adhesive type, indicating insufficient interfacial bonding and a weak adhesive performance.

In the present study, the SBS values measured for the PEP group (27.33 ± 2.87 MPa) were above the clinically acceptable threshold and consistent with those reported in previous [32]. According to the current findings, this bond strength did not differ significantly from that of Co–Cr frameworks subjected to appropriate surface conditioning followed by primer application (*p* < 0.05). Similarly, Jassim and Jaber [33] reported no statistically significant difference in SBS values when comparing sulfuric acid-etched PEEK to airborne-particle abraded Co–Cr frameworks. In MP and PEP groups, predominantly cohesive and mixed failure modes were observed, indicating the formation of a stronger and more durable bond between the substrates and PMMA.

A distinctive aspect of the present study was the integration of additional plasma and laser treatments into an already established surface conditioning protocol, aiming to further improve bond strength beyond previously reported values. However, contrary to expectations, the SBS value measured for the PEP-L group (21.07 ± 1.74 MPa) was lower than that of the PEP group without laser treatment (27.09 ± 1.53 MPa). Despite this numerical difference, the variation was not statistically significant (*p* > 0.05). At 20,000× magnification, fused pit borders, amorphous matrix zones, and contraction rings were clearly visible, indicating substantial morphological degradation following laser application on acid-etched surfaces (Figure 7). These SEM findings demonstrate a loss of microstructural continuity and suggest impaired interfacial adaptation due to surface over-processing. Notably, this is not a hypothetical assumption but a directly observed phenomenon in our study. Supporting this, a previous study. [34] reported that laser-created micro-retentions on the PEEK surface, when followed by sulfuric acid etching, did not result in a significant improvement in bond strength; in fact, lower SBS values were observed compared to acid etching alone. The authors attributed this to limited resin penetration into laser-induced microchannels, a hypothesis that our SEM data now visually confirms. In contrast, the sulfuric acid-treated group (PEP) exhibited a more consistent topography, characterized by submicron-scale pits and acid-induced porosities that enhanced surface activation without causing extensive structural collapse (Figure 5). These features closely resemble previously reported SEM findings of sulfuric acid-etched PEEK, which similarly demonstrate microporous textures favorable for resin infiltration and micromechanical retention [35,36].

The inconsistent findings reported in the literature highlight the need for a more nuanced understanding of how laser parameters interact with material-specific properties. In this context, the present study contributes valuable insight by systematically examining the potential—and limitations—of laser surface treatments within a well-established protocol [29,37]. Although laser treatment can be effective under certain conditions, it did not provide additional benefit within the acid–primer protocol used in this study. Both PEP and PEP-PL groups primarily exhibited cohesive and mixed failures, consistent with their relatively high SBS values. In contrast, the PEP-L group showed a higher rate of adhesive failures, aligning with its lower bond strength. Interestingly, as illustrated in Figure 3, adhesive failures were also observed in groups with high SBS values, indicating that fracture type alone may not reliably reflect bonding effectiveness, especially in protocols involving laser treatment [38,39,40] (Figure 3).

Plasma treatment enhances bond strength by creating chemically active functional groups on the PEEK surface; however, its effectiveness may vary depending on the application parameters. Previous studies have reported lower SBS values (8–15 MPa) when plasma treatment was followed by the use of MMA-based monomers alone, compared to the values obtained in the current study. Additionally, the SBS values obtained after aging were found to decrease further [41]. From this perspective, it can be concluded that plasma treatment alone is insufficient for PEEK surfaces. In a similar study, Schwitalla et al. performed sandblasting on PEEK surfaces prior to plasma treatment, and with this combination, they achieved high SBS values (~34.9 MPa), which aligns with the findings of the current study [42].

SEM analysis of the plasma-treated PEEK specimens supports this interpretation. At 10,000× magnification (Figure 8), the surface exhibited a uniform nano-textured topography with shallow dimple-like features and no signs of thermal deformation. At higher magnification (Figure 9), these characteristics appeared more defined, revealing ravine-like surface irregularities without matrix disruption or fiber damage. The preserved structural integrity and lack of deep porosity confirm the superficial and non-invasive nature of plasma modification, helping to explain its limited contribution to bond enhancement when potent chemical etching is already employed [41].

On the other hand, according to the results of the present study, no significant difference was observed in the bond strength between PEEK surfaces treated with acid and primer alone (PEP) and those treated with plasma in addition to the acid and primer application (PEP-PL) when bonded to PMMA (*p* > 0.05). Collectively, these findings challenge the presumed universal benefit of plasma treatment, revealing that when a potent chemical agent like sulfuric acid is already employed, plasma contributes no meaningful enhancement, underscoring a nuanced but clinically relevant insight that has not been explicitly stated in the prior literature. In the literature, the fracture patterns observed following bond strength tests conducted after plasma treatment alone are predominantly of the adhesive type, which supports the low SBS values reported [41]. Although studies involving primer application following plasma treatment tend to show a shift toward mixed-type fracture patterns, the current study differs in that the majority of the observed failures were cohesive in nature [43,44]. Although some studies have explored the potential of plasma treatment in combination with other surface treatments to enhance SBS, investigations focusing on the resulting fracture behavior remain scarce. In the present study, not a single purely adhesive failure was observed in the PEP-PL group; yet, intriguingly, this pattern mirrored the results of the PEP group. This indicates that plasma treatment did not contribute any additional benefit in altering failure mode either, a finding that, to the best of our knowledge, has never been explicitly reported in the existing literature.

Given the absence of direct evidence on the bond strength between FRC Trinia and PMMA in the current literature, this study deliberately adopted surface treatment protocols—airborne-particle abrasion and silica coating (CoJet)—that are not only backed by experimental data but are also widely trusted in clinical workflows [45,46]. Rather than exploring theoretical combinations, the aim was to strengthen real-world applicability by leveraging proven techniques to optimize bonding on the Trinia surface [47]. Accordingly, the shear bond strength (SBS) of treated Trinia specimens was systematically compared to that of a conventional metal control group to provide meaningful insights for clinical translation Comparable SBS values (~16.5–17.4 MPa) were observed in the groups treated with CoJet and primer (TCP), airborne-particle abrasion and primer (TAP), airborne-particle abrasion combined with laser (TAP-L), and airborne-particle abrasion combined with plasma (TAP-PL), with no statistically significant differences among them. In contrast, the SBS value measured in the metal control group (MP) (~28 MPa) was significantly higher than those of the Trinia groups (*p* < 0.05). SEM analysis revealed that, aside from silica deposition in the TCP group, the surface morphologies of TAP and TCP appeared largely similar; laser treatment did not induce dramatic topographical alterations as observed in PEEK specimens, and plasma application failed to produce any noticeable increase in surface roughness. These observations are morphologically consistent with previously reported SEM images of Trinia surfaces treated with airborne-particle abrasion and silica coating [45,48]. Nevertheless, the mean SBS values obtained from all Trinia groups exceeded the clinically accepted minimum threshold of 10 MPa, indicating that the applied surface treatments provided adequate bond strength for practical use. Due to the limited number of studies directly evaluating the bond strength between Trinia and PMMA surfaces, the present findings offer a meaningful and novel contribution to the field. What makes this gap even more surprising is the fact that Trinia is one of the few CAD/CAM framework materials with proven clinical reliability, yet it remains remarkably underrepresented in the literature. This discrepancy underscores the need for more focused investigations into materials that are not only accessible, but also clinically impactful.

In the study conducted by Meriç et al. [49], a fiber-reinforced composite base material demonstrated comparable bond strength to acrylic denture teeth without any surface treatment, with a mean SBS value of 19.3 ± 5.6 MPa, which was similar to that achieved by the conventional heat-polymerized PMMA base material (17.4 ± 5.2 MPa). Although the number of in vitro studies directly evaluating the bond strength between Trinia and PMMA is limited, several clinical reports have demonstrated successful long-term outcomes for prostheses fabricated using this material combination [50]. This discrepancy highlights a critical lag in the in vitro validation of a material that is already being successfully used in clinical practice. In the present study, the measured SBS values and the predominance of mixed-type failures observed after fracture testing indicate that, when appropriate surface treatments are applied, the bond between PMMA and the Trinia framework can reach clinically acceptable—and potentially durable—levels. While further investigations are warranted to explore a wider range of surface protocols and PMMA formulations, the present findings offer a strong foundation for the clinical integration of this material combination. By addressing a notable gap in the literature, this study not only supports the viability of Trinia in prosthetic applications but also encourages a shift toward evidence-based adoption of emerging CAD/CAM materials in daily clinical practice.

## 5. Conclusions

In this study, the effects of different surface treatment protocols on the bond strength between high-performance framework materials and heat-polymerized PMMA were evaluated, and the findings are summarized below:

1. The highest bond strength values were obtained between Co–Cr metal frameworks and PMMA, as well as in PEEK–PMMA combinations treated with sulfuric acid and primer, and those additionally subjected to plasma activation.
2. In specimens where laser irradiation was applied prior to sulfuric acid etching on PEEK surfaces, bond strength values were lower compared to acid-etched counterparts, suggesting that the additional thermal modification may have adversely affected the integrity of the bonding interface.
3. In Trinia specimens, the surface modification achieved through tribochemical silica coating or airborne-particle abrasion followed by primer application provided sufficient bond strength; however, the addition of plasma or laser treatments to these surface protocols did not result in any further improvement in bonding quality.
4. Fiber-reinforced composite (FRC) CAD/CAM frameworks such as Trinia demonstrated acceptable bonding performance to PMMA when appropriate surface treatments were applied, supporting their clinical viability as alternatives to metal-based frameworks.

These findings highlight the importance of selecting simplified, material-specific surface preparation protocols to ensure clinical success. Furthermore, energy-based surface modifications such as plasma or laser may not always enhance bonding and should be carefully considered based on the material type. In an era where CAD/CAM systems are becoming standard in prosthetic workflows, the potential of bio-compatible, esthetically favorable FRC materials like Trinia should not be overlooked.

## Figures and Tables

**Table 1:** Materials used in study.

Material Type	Product Name	Composition/Function	Manufacturer
Framework material	BioHPP	PEEK reinforced with 20–30% ceramic fillers (Al_2_O_3_, ZrO_2_)	Bredent GmbH & Co. KG, Senden, Germany
	Trinia™	Glass fiber-reinforced polymer composite	Bicon LLC/Harvest Dental, Boston, MA, USA
	Wirobond^®^ C+	Co 61%, Cr 25%, Mo 5%, Si 1.6%, Mn 0.9%, Fe < 1%	GmbH & Co. KG, Bremen, Germany
Surface agent	Scotchbond Universal	MDP, silane, Bis-GMA, ethanol	3M ESPE, St. Paul, MN, USA
	Visio.Link	MMA-based primer for PEEK/BioHPP	Bredent GmbH & Co. KG, Senden, Germany
Abrasive	Cobra	Aluminum oxide, 110 µm/Surface treatment for Trinia	Renfert GmbH, Hilzingen, Germany
Chemical agent	Merck (Sigma-Aldrich)	Sulfuric Acid (98%)/Surface treatment for BioHPP	Sigma-Aldrich, Saint Louis, MO, USA
Veneering material	Trevalon	Heat-cured polymethyl methacrylate	Dentsply Sirona, Gurgaon, India
Silica-coated abrasive	CoJet™ Sand	Silica-coated Al_2_O_3_ particles (30 µm)/tribochemical surface treatment of Trinia for silanization	3 M ESPE, Bayern, Germany
Fiber laser system	YLP Series	Ytterbium-doped fiber laser (1064 nm)/treatment for microstructuring of Trinia and BioHPP	IPG Photonics, Marlborough, MA, USA
Plasma system	PlasmaPrep III	Cold plasma treatment/surface activation of Trinia and BioHPP to enhance bonding.	IPG Photonics Corporation, Oxford, MA, USA
Abrasive	Cobra	Aluminum oxide particles (50 µm)/fine surface roughening of PMMA to improve adhesion	Renfert GmbH, Hilzingen, Germany
Surface conditioner	Trevalon	Methyl methacrylate (MMA)/surface swelling of heat-cured PMMA to enhance chemical bonding	Dentsply Sirona, Gurgaon, India
Index mold	Elite HD+ Putty	Vinyl polysiloxane for standardized cylinder shaping	Zhermack, Rovigo, Italy

**Table 2:** Mechanical and thermal properties of tested framework and veneering materials.

Material	Type	Flexural Strength (MPa)	Elastic Modulus (GPa)	Glass Transition Temperature (°C)	Thermal Conductivity (W/m·K)
BioHPP	PEEK reinforced with 20–30% ceramic fillers (Al_2_O_3_, ZrO_2_)	140–170	3–4	~143–150	~0.29
Trinia™	Glass fiber-reinforced polymer composite	280–350	15–18	~120–130	~0.30
Wirobond^®^ C+	Cobalt–chromium (Co 61%, Cr 25%, Mo 5%)	800–1000	~200	N/A (metallic material)	~14–16
Trevalon	Heat-polymerized polymethyl methacrylate (PMMA)	70–90	2.5–3.5	~105–110	~0.20

**Table 3:** Group classification with associated surface treatment codes.

Group Code	Surface Treatment Description
MP	Co-Cr; Sandblasting + Metal Primer
PK	BioHPP; Untreated (Control)
PEP	BioHPP; Sulfuric acid (98%) + Primer (Visio.link)
PEP-PL	BioHPP; Sulfuric acid (98%) + Primer + Plasma
PEP-L	BioHPP; Sulfuric acid (98%) + Primer + Laser
TCP	Trinia; Cojet + Primer
TAP	Trinia; Sandblasting + Primer
TAP-PL	Trinia; Sandblasting + Primer + Plasma
TAP-L	Trinia; Sandblasting + Primer + Laser

**Table 4:** Mean ± SD values and post hoc comparisons of SBS.

Group	Mean ± SD (MPa)	N
MP	27.99 ± 1.50 ᶜ	15
PK	3.92 ± 1.17 ᵃ	15
PEP	27.09 ± 1.53 ᶜ	15
PEP-L	21.07 ± 1.74 ᵇᶜ	15
PEP-PL	27.85 ± 1.26 ᶜ	15
TCP	16.68 ± 0.98 ᵇ	15
TAP	16.54 ± 0.44 ᵇ	15
TAP-L	16.98 ± 1.62 ᵇ	15
TAP-PL	17.38 ± 0.59 ᵇ	15

Note: Different superscript letters indicate statistically significant differences between groups (*p* < 0.05). Groups sharing the same letter are not significantly different.

## Data Availability

The original contributions presented in this study are included in the article. Further inquiries can be directed to the corresponding author.
